# Bacterial Whack-a-Mole: Reconsidering the Public Health Relevance of Using Carbadox in Food Animals

**DOI:** 10.1128/mBio.01490-17

**Published:** 2017-09-26

**Authors:** Lance B. Price

**Affiliations:** Milken Institute School of Public Health, George Washington University, Washington, DC, and Division of Pathogen Genomics, Translational Genomics Research Institute, Flagstaff, Arizona, USA

**Keywords:** agriculture, antibiotic, antibiotic resistance, antimicrobial, carbadox, health policy, hog, livestock, mobile elements, pig, stewardship, swine

## Abstract

Carbadox is an antibiotic used to control dysentery and promote growth in swine in the United States; however, the drug also causes tumors and birth defects in laboratory animals. Despite this and because the drug has no analogs in human medicine, it is not considered “medically important” and can be used in livestock without veterinarian oversight. In their recent study, T. A. Johnson et al. (mBio 8:e00709-17, 2017, https://doi.org/10.1128/mBio.00709-17) demonstrated that carbadox has profound effects on the swine gut microbiome, including the induction of transducing phage carrying tetracycline, aminoglycoside, and beta-lactam resistance genes. In swine production, carbadox can be used in conjunction with other antibiotics (e.g., oxytetracycline) that could fuel the emergence of strains carrying phage-encoded resistance determinants. Johnson et al.’s findings underscore the potential unforeseen consequences of using antibiotics in livestock production and call into question our current methods for classifying whether or not a veterinary drug has relevance to human health.

## COMMENTARY

Antibiotic resistance has finally captured the attention of the public and policymakers around the world. For years, only a few outspoken scientists were ringing alarms about the clashing trends of increasingly resistant bacterial infections and the decreasing pace of antibiotic development (1, 2). Even fewer people were discussing the potential public health risks of antibiotic use in livestock production (3, 4). That began to change with a number of high-profile reports that described the emerging antibiotic resistance crisis in terms that the general public could understand and highlighted the potential risks of using antibiotics in food animals (5, 6). These reports have been accompanied by heated policy debates where stakeholders have sought to define antibiotic applications into categories, such as “necessary versus unnecessary” or “therapeutic versus nontherapeutic” and have tried to categorize antibiotics by their relative importance to human medicine (7). A recent *mBio* study by Johnson et al. (8) investigating the impact of carbadox on the swine gut microbiome provides an important example of how even antimicrobial agents with no foreseeable utility in human medicine may pose a public health threat when used in food animals.

U.S. policymakers are not known for their quickness, so when the U.S. Food and Drug Administration (FDA) finally took steps to eliminate the most egregious agricultural use of antibiotics—growth promotion—consumers were already demanding more, and the market responded with several big-name retailers announcing new, more-restrictive antibiotic use policies for their suppliers. One of the terms that has made its way into the lexicon of agricultural antibiotic stewardship is “medically important” as an adjective describing certain antibiotics. Several companies have announced that they will prohibit the use of “medically important antibiotics” for routine disease prevention. This naturally leads to the question of which antibiotics are medically important. After all, there are drugs that are used exclusively in livestock, such as the third-generation cephalosporin ceftiofur or the fluoroquinolone enrofloxacin. These two drugs, ceftiofur and enrofloxacin, are never used in human medicine, but bacteria that evolve resistance to these drugs are also resistant to their human medicine analogs, ceftriaxone and ciprofloxacin, respectively. Large-scale, real-world studies have demonstrated that veterinary use of these antimicrobials can lead to resistant infections in people (9, 10). To their credit, some companies have released policies that specifically limit the use of antimicrobials belonging to the same classes that are used in human medicine. Beyond these shared analogs, there are those that belong to antibiotic classes that are used exclusively in food animals, including ionophores and the quinoxaline-di-*N*-oxide, carbadox. These antibiotics seem to pose minimal risk to human health, at least on the surface.

Carbadox has a checkered history in livestock production. Carbadox is used to treat bacterial enteritis and to promote growth in swine, but the drug is also a known teratogen and a suspected carcinogen. Because it is not considered medically important in human medicine, the drug can be used in livestock without veterinarian oversight. While its potential carcinogenicity in humans has not been assessed by the U.S. Environmental Protection Agency’s Integrated Risk Information System (IRIS) program or the International Agency for Research on Cancer, it has been banned from food animal production in the European Union and Australia based on its potential risk to people. In April 2016, the FDA started procedures to withdraw approval for carbadox in U.S. food animals if the drug sponsors are unable to prove that it does not pose a cancer risk to humans (11).

**FIG 1:**
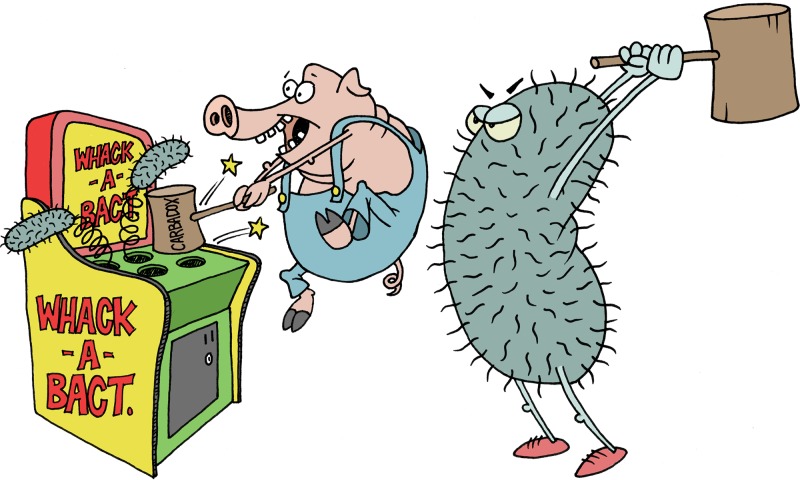
Bacterial whack-a-mole. Using carbadox in swine production may help prevent some bacterial infections, but it may also lead to the emergence of bacteria that are resistant to antibiotics used in human medicine.

However, the study by Johnson et al. (8) indicates that using carbadox in food animals may not only increase the risk for cancer and birth defects, but it may also fuel the transmission of phage-encoded antimicrobial resistance genes. Carbadox is genotoxic and mutagenic and thus a potent inducer of the SOS pathway and prophage. By prospectively analyzing the gut microbiomes of swine fed standard doses of carbadox and swine given unmedicated feed, the authors showed an acute induction of prophage and transfer of phage-encoded antibiotic resistance genes. Paradoxically and relevant to the question of whether carbadox should be considered medically important, some of the transferred genes coded for resistance to antibiotic classes that are commonly used in human medicine, including tetracyclines, aminoglycosides, and beta-lactams. While transmission of these genes was associated with carbadox treatment, they did not increase in absolute abundance during the treatment period. However, the study was conducted using carbadox alone, while in actual production settings, the drug would frequently be accompanied or immediately followed by other antibiotics. For some applications, the drug sponsor actually recommends using carbadox in conjunction with oxytetracycline, which would likely fuel the expansion of bacterial populations that acquire tetracycline resistance genes as a result of the carbadox-induced phage transmission (12). Future studies will have to be conducted to determine whether carbadox acts synergistically with other drugs to encourage the rapid emergence of pathogens resistant to the antibiotics administered along with carbadox (Fig. 1).

With the FDA’s action pending, the fate of carbadox in U.S. food animal production is unclear; nonetheless, this study (8) underscores the risks for unintended consequences when using antimicrobials in livestock and should force us to reconsider how we evaluate the human health relevance of the use of any antimicrobial. In an attempt to compromise with food animal producers and drug companies, some in the public health community (including myself) have agreed to classify ionophores as medically unimportant. However, many of us have done so with fears that these drugs may pose unforeseen risks, such as those described by Johnson et al. (8). Furthermore, regardless of what happens with carbadox in the United States, there is a growing demand for antimicrobials for food animal production in the developing world (13), where the potential for this drug to be used in concert with antimicrobials of critical importance to human health is high. Thus, this study has global relevance and should be taken into consideration as the developing world tries to meet their growing demand for animal protein while protecting their citizens from the growing threat of multidrug-resistant infections.
